# Genetic Variants of the Receptor Activator Nuclear of κB Ligand Gene Increase the Risk of Rheumatoid Arthritis in a Mexican Mestizo Population: A Case–Control Study

**DOI:** 10.3390/genes15070907

**Published:** 2024-07-11

**Authors:** Nava-Valdivia Cesar Arturo, Gamez-Nava Jorge Ivan, Contreras-Haro Betsabe, Perez-Guerrero Edsaul Emilio, Esparza-Guerrero Yussef, Rodriguez-Jimenez Norma Alejandra, Gonzalez-Heredia Tonatiuh, Villagomez-Vega Alejandra, Nuño-Arana Ismael, Totsuka-Sutto Sylvia Elena, Ponce-Guarneros Juan Manuel, Jacobo-Cuevas Heriberto, Alvarez-Ayala Efren Gerardo, Gonzalez-Lopez Laura, Saldaña-Cruz Ana Miriam

**Affiliations:** 1 Departamento de Microbiología y Patología, Centro Universitario de Ciencias de la Salud, Universidad de Guadalajara, Guadalajara 44340, Mexico; 2 Programa de Doctorado en Farmacología, Departamento de Fisiología, Centro Universitario de Ciencias de la Salud, Universidad de Guadalajara, Guadalajara 44340, Mexico; 3 Instituto de Terapéutica Experimental y Clínica, Departamento de Fisiología, Centro Universitario de Ciencias de la Salud, Universidad de Guadalajara, Guadalajara 44340, Mexico; 4 Unidad de Investigación Biomédica 02, UMAE, Hospital de Especialidades, Centro Médico Nacional de Occidente, IMSS, Guadalajara 44349, Mexico; 5 Departamento de Ciencias Biomédicas, Centro Universitario de Tonalá, Universidad de Guadalajara, Guadalajara 45400, Mexico; 6 Instituto de Investigación en Ciencias Biomédicas, Centro Universitario de Ciencias de la Salud, Universidad de Guadalajara, Guadalajara 44340, Mexico

**Keywords:** rheumatoid arthritis, genetic variant, RANK ligand

## Abstract

The Receptor Activator Nuclear of κB Ligand (RANKL) plays an important function in immune responses, activating osteoclast cells and unchanged bone resorption, which in turn leads to bone erosion and inflammation. Genetic variants in the promoter region of the RANKL gene could lead to a higher risk of rheumatoid arthritis (RA). Objective: To assess the association of *rs9533155* (-693C>G) and *rs9533156* (-643T>C) genetic variants with RA risk. Methods: A case–control study was carried out. A total of 94 patients with RA (RA group) and 134 subjects without any rheumatologic disease (control group) were included. Genetic DNA was extracted from peripheral white blood cells (leukocytes). Genetic variant *rs9533155* (-693C>G) was screened by an approach based on Polymerase Chain Reaction–Restriction Fragment Length Polymorphism (PCR-RFLP), while *rs9533156* (-643T>C) was screened using quantitative polymerase chain reaction (qPCR) with TaqMan probes. RANKL serum levels were measured by ELISA. Results: For *rs9533155* (-693C>G), the polymorphic homozygous genotype frequencies (CC) were higher in the RA group (*p* = 0.006). Individuals carrying the risk genotype presented higher levels of serum RANKL. Carriers of the polymorphic homozygous genotype in the dominant model (CC vs. CG + GG) had an increased risk of developing RA (OR: 1.8, 95% CI 1.04 to 3.1). No association between *rs9533156* (-643T>C) and the haplotypes with RA risk was observed. Conclusion: The *rs9533155* (-693C>G) genetic variant exhibits a potential role in RA risk. The studied population had no association with the *rs9533156* (-643T>C) genetic variant.

## 1. Introduction

Rheumatoid arthritis (RA) is a chronic inflammatory disease characterized by erosive joint damage, deterioration of bone mass, and cartilage damage related to the immune response, which consequently impacts health-related quality of life [1]. The etiology of RA still needs to be fully elucidated. However, it has been estimated that genetic factors may influence 50% of RA disease development [2]. The human leukocyte antigen (HLA) class II locus is the key factor that increases susceptibility to RA development. About 150 loci have been studied for the susceptibility to developing RA [3]. Rheumatoid arthritis is a phenotypically heterogeneous disease, caused by genetic variants that have been associated with different clinical presentations of RA. For instance, it has been reported that there is an association between HLADRβ-11 and the development of antidrug antibodies in patients with rheumatic diseases, including RA [4]. Moreover, another study found that positive HLA-DRB1*11 may protect against risks related to mobility, physical impairment, dexterity, household activities, activities of daily living, social activity, pain, anxiety, and depression [5].

In the pathogenesis of RA, osteoclasts significantly contribute to the development of articular cartilage inflammation and destruction [6] and are additionally involved in bone resorption and erosion [7]. RANKL, a member of the tumor necrosis factor-related cytokines (TNF-α) superfamily, is coded by a single gene: the tumor necrosis factor ligand superfamily member 11 (*TNFSF11*) [8]. This protein is expressed in osteocytes, osteoblasts, stromal cells, natural killer, and activated T and B cells [7,9]. It stimulates osteoclast differentiation, activation and survival from monocyte/macrophage lineage precursor cells and is among the factors that enhance the differentiation from immature osteoclasts to mature osteoclasts [10,11]; consequently, RANKL overexpression leads to osteoclast differentiation with an increase in bone resorption [12]. Furthermore, an essential role of RANKL in the immune response modulation has been proposed. This is due to its role in regulating dendritic cells expressing receptor activator of NF-κB (RANK) in their membrane [13], where in the first instance, RANK was recognized as a factor that promotes dendritic cell survival [13,14]. RANK/RANKL binding promotes the increased ability of dendritic cells to activate naive T cell proliferation. Thus, RANKL has been proposed to promote autoantigen presentation in patients with RA [15].

The interaction between RANK and RANKL is also involved in medullary thymic epithelial cell maturation and the negative selection of autoreactive T cells. Thus, it has a crucial role in the immune cell tolerance and, thereby, the generation of autoimmunity [16,17].

In RA, the overexpression of RANKL linked to RANK promotes the activation of osteoclast cells and unchanged bone resorption, a key step in the pathogenesis of erosion and inflammation [18]. However, the tendency to overexpress and increase the functioning of RANKL is induced by many mechanisms, including genetic variants of the RANKL gene located in the promoter region, which may significantly influence the transcription and the RANKL protein levels. 

Previous studies have analyzed the association between genetic variants of the *RANKL* gene and RA risk [19,20,21,22,23,24,25]. It is essential to investigate sequence variations in the promoter region that may impact the transcription of the *RANKL* gene, such as the SNPs -643 and -693. However, it is important to note that only Assman et al. evaluated *rs9533156* of the *RANKL* gene, which was found to be a protective factor against RA in the German population [17]. The gap in our knowledge becomes more apparent when we consider the lack of information in the Mexican population about the *rs9533155* (-693C>G) and *rs9533156* (-643T>C) genetic variants of the *RANKL* gene and the risk of RA.

Therefore, the aim of this study is to evaluate the association of *rs9533155* (-693C>G) and *rs9533156* (-643T>C) *RANKL* gene genetic variants with RA risk. 

## 2. Materials and Methods

The research design employed in this study was a case–control investigation involving two study groups from Mexican Mestizo ethnicity, defined by the Mexican National Institute of Anthropology and History (“INAH”) as “individuals who were born in Mexico, there generation including their own and were descendants of the original autochthonous inhabitants of the region and individuals who were mainly Spaniards” [26]. The RA group included 94 patients with rheumatoid arthritis (RA) who met the eligibility criteria established by the American College of Rheumatology (ACR) in 1987 [27] and were of female sex and were Mexican Mestizo. The control group comprised 134 subjects of similar age, who were selected from the Clinical and Experimental Therapeutic Institute. The selection process for the study excluded individuals with chronic infectious diseases, including hepatitis B, C, or HIV infections; chronic renal failure; transaminasemia with values exceeding twice the normal range; and cancer.

### 2.1. Clinical Assessments

The researchers conducted structured interviews and physical examinations of all patients included in the study to collect information on their sociodemographic and disease characteristics as well as their current therapy. This involved assessing patients’ clinical and sociodemographic features. Menopause was defined as the absence of menstruation for at least a year caused by a decrease in female sex hormones due to natural causes, excluding diseases or surgical menopause. Disease activity was assessed by a trained researcher using the Disease Activity Score-28 (DAS28) [28], which includes (1) 28 swollen joint counts, (2) 28 tender joint counts, (3) a global health index perceived by the patient, and (4) erythrocyte sedimentation rate (ESR) [29]. Functioning was measured using the Health Assessment Questionnaire-Disability Index (HAQ-Di) [30].

### 2.2. Laboratory Assessments

In this study, antibody determination was performed using an enzyme-linked immunosorbent assay (ELISA). To determine the levels of anti-cyclic citrullinated peptide antibody (anti-CCP) and anti-mutated citrullinated vimentin antibody (anti-MCV), the following commercial kits were used: Euroimmun, Medizinische Labordiagnostika, Germany and Orgentec Di-agnostika GmbH, Mainz, Germany, respectively. The cutoff points for positivity were determined based on the manufacturer’s recommendations: (a) CRP > 10 mg/L, (b) RF > 20 IU/mL, and (c) anti-CCP > 5 RU/mL and anti-MCV > 20 U/mL. The cutoff points for anti-CCP antibodies were validated in a previous study conducted in our laboratory [31]. In addition, a commercial ELISA kit (R&D Systems Inc. Minneapolis, MN, USA) was used for the determination of tumor necrosis factor-α, and a commercial ELISA was used for the serum determination of RANKL (Biovendor Laboratorni Medicina A. S., Brno, Czech Republic) (pmol/L, with a sensitivity of 0.4 pmol/L). Nephelometry was used to determine the serum C-reactive protein (CRP) and rheumatoid factor (RF).

### 2.3. Genotype Analysis of the RANKL Gene

Genotype analysis of the RANKL gene was performed on 228 subjects, DNA was extracted from leukocytes in peripheral blood samples using the modified Miller technique [32]. DNA was quantified using a Nanodrop Genomic, diluted in Tris-EDTA buffer to 20 ng/μL, and placed in 200 μL propylene cryotubes. The *rs9533155* (-693C>G) genotype was screened using Polymerase Chain Reaction–Restriction Fragment Length Polymorphism (PCR-RFLP). The 552 bp PCR product was incubated at 37 °C with 10U of BsaJI (New England BioLabs, Ipswich, MA, USA) restriction endonuclease, which cuts the PCR product into two fragments of 356 and 196 bp in the presence of the C (Silvestre allele). The resulting fragments were visualized by electrophoresis on 6% polyacrylamide gels followed by silver staining. Genotyping of the *rs9533156* (-643T>C) genetic variant was performed using quantitative polymerase chain reaction (qPCR) with TaqMan probes [33]. TaqMan ID protocols were followed according to the manufacturer’s instructions, and the StepOne Real-Time PCR system was employed for this purpose (Applied Biosystems, Waltham, MA, USA). The haplotypes formed by the four RANKL variants were inferred by considering the following order of alleles (from centromere to telomere): *rs9533155* (allele 1 = C, allele 2 = G) and *rs9533156* (allele 1 = T, allele 2 = C). For qualitative variables, frequencies (percentages) were calculated, whereas for quantitative variables, the median and ranges were determined.

## 3. Statistical Analyses

Allele frequencies were determined by counting observed genotype frequencies. Data normality was determined using the Kolmogorov–Smirnov test. The Mann–Whitney U test was used to compare medians, while the chi-square test or Fisher’s exact test was used to compare proportions. The Kruskal–Wallis test and post hoc analysis (using the T3-Dunnet test) were performed to compare the serum levels of RANKL for each genotype of *rs9533155* and *rs9533156*. Spearman correlation coefficients were used to determine the strength of the association between RANKL and other clinical numeric variables. Odds ratios (ORs) between cases and controls were calculated, along with 95% confidence intervals (95% CIs). The genetic models adopted in the present study were dominant (CC vs. CG + GG) and recessive (CC + CG vs. GG) for *rs9533155* and (TT vs. TC + CC) as dominant and (TT + TC vs. CC) as recessive for *rs9533156*. Hardy–Weinberg equilibrium (HWE) in control subjects was determined using the chi-square test. Statistical significance was set at *p* ≤ 0.05, and the data were analyzed using SPSS software (version 23.0; SPSS Inc., Chicago, IL, USA). Odds ratios (ORs) and their confidence intervals (95% CI) were obtained using EPI-INFO version 7.2 (Epi Info TM, Atlanta, GA, USA). The haplotypes were inferred using Arlequin software version 2007 (University of Berne, Bern, Switzerland), and the Hardy–Weinberg equilibrium was evaluated. Figures were generated using GraphPad Prism software (Ver 8.0.1, GraphPad Software, Inc., La Jolla, CA, USA).

## 4. Results

Table 1 presents the clinical characteristics of the 94 patients with RA. These patients had a median DAS28 score of 2.9 (2.3–4.1), and 75.5% were treated with synthetic DMARDs. Serum RANKL levels were 393 (200–861) pmol/L.

In the comparison between patients with RA and the control group, no statistically significant differences were observed in age (64 vs. 61, *p* = 0.9). No association of serum RANKL levels with the presence of disease activity (*p* = 0.25) was observed; similarly there was no association with seropositivity to anti-CCP (*p* = 0.85) and anti-MCV (*p* = 0.09). No correlation was observed between RANKL and anti-CCP and TNF-α (rho = −0.05, *p* = 0.66; and rho = −0.12, *p* = 0.25, respectively), in contrast to anti-MCV, where a correlation was observed (rho = 0.32, *p* = 0.002) [data not shown in tables].

Table 2 compares genotypes and allele frequencies of the genetic variants of *rs9533155* and *rs9533156* of the RANKL gene in patients with RA and the control group. The genotype distributions of the polymorphisms in the control group were consistent with HWE (*p* > 0.05). For the *rs9533155*, wild homozygous genotype C/C had a higher frequency in RA compared with the control group (46.8% vs. 32.8%), whereas mutated homozygous genotype G/G was less frequently observed in patients with RA (22.3% vs. 42.6%). In the dominant and recessive genetic models, when carriers of the C/C genotype are compared with carriers of the C/G or G/G genotype (dominant model), the risk of RA increases (OR: 1.80, 95%CI 1.04 to 3.10). No statistically significant difference in the risk of RA was observed with the recessive model. In addition, no statistically significant difference was observed between the allele frequencies. In the comparison of genotype frequencies of the genetic variant *rs9533156*, the wild genotype T/T had a higher frequency in RA compared with the control group (44.7% vs. 34.3%), whereas the mutated homozygous C/C was less frequently observed in patients with RA (13.8% vs. 16.4%). In the genetic models, no differences were observed between the carriers of wild homozygote, heterozygote, or mutated homozygous genotypes for RA risk. Furthermore, no differences were observed between the haplotype constructions, which showed four groups: haplotype CT (referent), GC, GT, and CC. The highest frequency haplotype in patients with RA was CT with 48.9%, whereas the haplotype CC had a lower frequency at 9.6%; however, no association was observed between haplotypes and RA susceptibility in both polymorphisms (Table 2).

**Figure 1:**
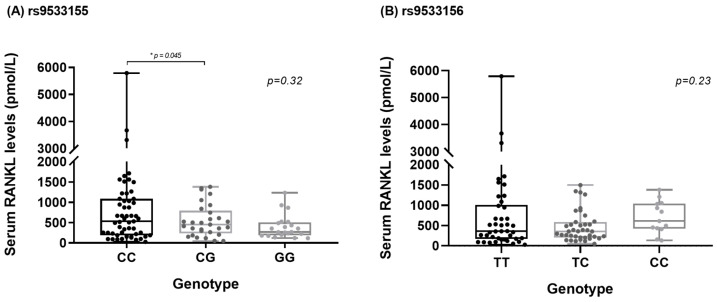
(**A**) Comparison of human serum sRANKL concentrations between single-nucleotide polymorphisms (SNP) *rs9533155* of *RANKL* gene. Data are presented as medians and ranges of picomole per liter of sRANKL serum levels (determined by ELISA), for CC (*n* = 44), CG (*n* = 29) and GG (*n* = 21) genotype groups, using the Kruskal–Wallis test. In a comparison between the genotypes in post hoc analysis using the T3-Dunnet test, the *p*-value was 0.045. (**B**) Comparison of human serum sRANKL concentrations between single-nucleotide polymorphism (SNP) *rs9533156* of *RANKL* gene. Data are presented as medians and ranges of picomole per liter of sRANKL serum levels (determined by ELISA), for TT (*n* = 42), TC (*n* = 39) and CC (*n* = 13) genotype groups, using the Kruskal–Wallis test.

Figure 1 shows the comparison of serum sRANKL levels between genotypes of polymorphism *rs9533155* and *rs9533156* of the *RANKL* gene in patients with RA. For *rs9533155*, higher sRANKL levels were observed in RA patient carriers of the C/C genotype compared with carriers of the C/G genotype (*p* = 0.04). No statistically significant difference in serum sRANKL levels was observed in RA patient carriers of the C/C genotype compared with carriers of the G/G genotype (*p* = 0.1). For *rs9533156*, no statistically significant difference was observed in serum RANKL levels between the genotypes. 

## 5. Discussion

Our results show that the dominant model (CC vs. CG + GG) of the *rs9533155* genetic variant of the *RANKL* gene confers risk for RA. In contrast, the *rs9533156* genetic variant does not increase the risk of RA in the Mexican Mestizo population. 

The *RANKL* gene is located on chromosome 13q14.11, which encodes a protein with 316 amino acids [34], and belongs to the tumor necrosis factor family, the crucial molecule mediating osteoclast development, activity, survival and consequently osteoclastogenesis. Additionally, it mediates immune response; it modulates B and T lymphocytes differentiation, upholds mature dendritic cells survival, promotes activation of monocytes, and participates in lymph node development and medullary thymic epithelial cells [17,35]. Increased serum RANKL levels are associated with bone resorption, suggesting a pivotal role in mediating bone erosion [36,37]. 

Some studies have explored, in various populations, the association between genetic variants of the *RANKL* gene and the risk of RA development [19,20,21,22,23,24,25]. Wu et al. 2004 reported a lack of association between other *RANKL* polymorphisms with erosive disease at baseline or an early radiographic joint damage progression rate [19]. On the other hand, in 2010, Tan et al. reported an association between the rs7984870 genetic variant and an early age at onset of RA seropositive (RF or anti-CCP positive) patients [18]. Additionally, three variants of the *RANKL* gene (rs7984870, rs7325635, rs1054016) showed a significant association with ACPA levels. The rs7325635 was related to erosions in patients with RA [23]. Moreover, Wielińska et al. observed that in both RANKL single nucleotide polymorphisms (rs7988338 G<A and rs7325635 G<A), GG homozygosity was significantly associated with the number of swollen joints, prior to and at the 12th week after therapy with anti-TNFα [24]. Nevertheless, to the best of our knowledge, our study is the first to explore the association between selected genetic variants and RANKL levels in Mexican patients with RA. 

Our results for *rs9533156* are similar to the findings of Assman et al., where no association between RA and this polymorphism was observed (OR: 0.79, CI95% 0.34 to 1.66, *p* = 0.14). Furthermore, it has been reported that other variants of the *RANKL* gene conferred risk for increased plasma soluble RANKL levels in patients with RA [21]. We found in our study that the -693C>G variant in the *TNFSF11* promoter region modifies the concentration of this protein, given that individuals carrying the risk genotype showed higher serum RANKL levels. In contrast, no differences were observed in serum RANKL levels with the -643T>C single nucleotide polymorphism. Previous studies have shown that sequence variations in the promoter region may impact the transcription of the *RANKL* gene. The SNPs -643 and -693 in the promoter region could modify the binding to transcription factors (TFs). They generate a greater binding, increasing gene expression and protein levels [34]. 

Wang et al. determined in their study through sequencing the *RANKL* gene that the gene repressor is located in the promoter region at positions -300 to -1000, where the genetic variants -693G>C and -643C>T are located. They showed that the genetic variant -693G>C significantly affects DNA–protein complex formation. They found no association with the genetic variant -643C>T. Moreover, the authors generated haplotypes with the genetic variant -693G>C and another variant -290T>C (rs9525641); the haplotypes they generated suppressed the activity of the promoter in a different way, which suggests that these SNPs may be functional [38]. 

In the present study, although -693G>C confers risk for RA, generating haplotypes with both genetic variants -693G>C and -643C>T yielded no significant differences. It is important to highlight the effect of each polymorphism on the phenotype, which is considerably low (1–3%), in order to explain the lack of differences [39]. Also, we only analyzed two genetic variants of the *RANKL* gene; hence, other genetic variants located in different sites could be related to RA development. 

On the other hand, RANKL is expressed by a large plethora of immunity cells, and consequently, it can be induced by different inflammatory factors such as interleukin 1, TNF- α, interleukin 1β, interleukin 6 and interleukin 17 [40,41]. 

High levels of TNF-α could generate greater osteoclastogenesis, as well as high serum levels of RANKL [42]; however, regarding the association between RANKL serum levels and TNF-α, no correlation was found, which demonstrates the effect of the genetic variant on the function of the promoter, apparently generating greater binding of TFs and greater production of RANKL, increasing osteoclast activation independently of pro-inflammatory cytokines.

Polymorphisms in the promoter region of the *RANKL* gene have been identified as potential drivers of changes in mRNA expression, which could lead to increased inflammation in patients with RA. It is speculated that functional SNPs in the promoter region of RANKL may enhance gene expression, but it is crucial to determine their impact on serum RANKL levels. An important consideration is the need for proteases in the production of soluble RANKL because it must be cleaved from the type II membrane protein [25].

However, our study has some limitations that must be considered. First, we did not measure mRNA expression, which has been reported as the direct effect of the presence of polymorphism. Second, the evaluation of RANKL levels was not carried out on a control group to identify the cut-off of RANKL in a population without RA. However, this aim was beyond the scope of the present study. Third, another limitation of this study was the design, as a case-control study cannot demonstrate causality. On the other hand, one of the strengths of this study is that, this is the first to analyze the association between RANKL genetic variants and the risk of RA in the Mexican population. Another strength is that we compared genetic variants of RANKL and the serum levels of the protein. This integral approach can provide a comprehensive assessment of the impact of the polymorphism on the development of the disease. 

## 6. Conclusions

In conclusion, the results obtained suggest the important role of the *rs9533155* (-693C>G) genetic variant in the pathogenesis of RA. These findings contribute to a better understanding of the genetic factors involved in the development of RA in the Mexican population. However, further research is needed to support these findings and to reinforce the biological role of genetic interactions involving RANKL in patients with RA.

## Figures and Tables

**Table 1:** Clinical characteristics of patients with rheumatoid arthritis.

Variable	*n* = 94
Age (years), *median (ranges)*	64 (55–68)
Alcoholism, *n (%)*	2 (2.1)
Smoking, *n (%)*	7 (7.4)
Sedentary lifestyle, *n (%)*	61 (64.9)
Disease duration (years), *median (ranges)*	12 (1–45)
DAS28-ESR score, *median (ranges)*	2.9 (2.3–4.1)
HAQ-DI score, *median (ranges)*	0.11 (0.00–0.67)
*Treatment*	
Synthetic Disease Modifying Anti-Rheumatic Drugs (cs-DMARDs, n (%)	71 (75.5)
Monotherapy with 1 cs-DMARD, *n (%)*	28 (29.8)
Combined therapy with ≥2cs-DMARDs, *n (%)*	43 (45.7)
Biologics, *n (%)*	2 (2.1)
Glucocorticoid, *n (%)*	69 (73.4)
Laboratory assessment	
Erythrocyte Sedimentation Rate (mm/h), *median (ranges)*	18 (18–32)
Rheumatoid Factor titers (UI/mL), *median (ranges)*	5 (0–18)
Anti-CCP2 (RU/mL), *median (ranges)*	20 (2–179)
Anti-CCP2 positive, *n (%)*	54 (57.4)
Anti-MCV (U/mL), *median (ranges)*	42 (8–334)
Anti-MCV positive, *n (%)*	55 (58.5)
Serum Tumor Necrosis Factor-α (pg/mL), *median (ranges)*	11 (4–22)
Serum sRANKL levels (pmol/L), *median (ranges)*	393 (200–861)

DAS28: Disease activity for 28 joints; HAQ-DI: Health Assessment Questionnaire-Disability Index; ESR: erythrocyte sedimentation rate; anti-CCP second generation (Anti-CCP2), anti-mutated citrullinated vimentin antibodies (anti-MCV); sRANKL: Soluble Receptor Activator for Nuclear Factor Kappa B ligand; qualitative variables were expressed in frequencies and quantitative variables in medians and ranges.

**Table 2:** Comparison of genotypes, alleles and haplotypes of the *RANKL* gene *rs9533155* and *rs9533156* in rheumatoid arthritis patients and controls.

	Rheumatoid Arthritis *n* = 94	Control Group *n* = 134	OR	95% CI	*p*	HWE *p* Value
*Genotype rs9533155 (C>G)*						
CC, *n* (%)	44 (46.8)	44 (32.8)	-	-		X^2^ = 3.07 *p* = 0.079
CG, *n* (%)	29 (30.9)	33 (24.6)	-	-	0.006	
GG, *n* (%)	21 (22.3)	57 (42.6)	-	-		
*Genetic models*						
Dominant model (CC vs. CG + GG)	44 vs. 50	44 vs. 90	1.80	1.04 to 3.10	0.03	
Recessive model (CC + CG vs. GG)	31 vs. 63	34 vs. 100	1.45	0.81 to 2.59	0.2	
*Allele, 2n*						
C, *n* (%)	117 (62.2)	121 (45.1)				
G, *n* (%)	71 (37.7)	147 (54.9)				
Genotype *rs9533156* (*T* > C)						
TT, *n* (%)	42 (44.7)	46 (34.3)	-	-		X^2^ = 0.127 *p* = 0.721
TC, *n* (%)	39 (41.5)	66 (49.3)	-	-	0.3	
CC, *n* (%)	13 (13.8)	22 (16.4)	-	-		
*Genetic models*						
Dominant model (TT vs. TC + CC)	42 vs. 52	46 vs. 88	1.54	0.89 to 2.66	0.06	
Recessive model (CC vs. TT + TC)	13 vs. 81	22 vs. 112	0.82	0.38 to 1.71	0.7	
*Allele*						
T, *n* (%)	123 (65.4)	158 (58.9)				
C, *n* (%)	65 (34.5)	110 (41.1)				
Haplotypes						
Haplotype 1 CT	46 (48.9)	60 (44.8)	1.17	0.62 to 2.19	0.6	
Haplotype 2 GC	25 (26.6)	38 (28.4)	1.00	-	-	
Haplotype 3 GT	14 (14.9)	21 (15.7)	1.01	0.44 to 2.36	0.9	
Haplotype 4 CC	9 (9.6)	15 (11.1)	0.91	0.35 to 2.40	0.8	

Qualitative variables are expressed in frequency and percentage. For genotype *rs9533155*, GG: wild homozygous genotype; CG: heterozygous genotype; CC: polymorphic homozygous genotype. For genotype *rs9533156*, CC: wild homozygous genotype; TC: heterozygous genotype; TT: polymorphic homozygous genotype. OR: odds ratio. 95% CI: confidence interval 95%. HWE: Hardy–Weinberg equilibrium. *p*-value as significant ≤ 0.05.

## Data Availability

The original contributions presented in the study are included in the article, further inquiries can be directed to the corresponding author.

## References

[1] Lin Y.-J., Anzaghe M., Schülke S. (2020). Update on the Pathomechanism, Diagnosis, and Treatment Options for Rheumatoid Arthritis. Cells.

[2] Radu A.-F., Bungau S.G. (2021). Management of Rheumatoid Arthritis: An Overview. Cells.

[3] Padyukov L. (2022). Genetics of rheumatoid arthritis. Semin. Immunopathol..

[4] Benucci M., Damiani A., Gobbi F.L., Bandinelli F., Infantino M., Grossi V., Manfredi M., Noguier G., Meacci F. (2018). Correlation between HLA haplotypes and the development of antidrug antibodies in a cohort of patients with rheumatic diseases. Biol. Targets Ther..

[5] Bandinelli F., Benucci M., Salaffi F., Manetti M., Infantino M., Damiani A., Manfredi M., Grossi V., Matucci A., Gobbi F.L. (2021). Do new and old biomarkers of early undifferentiated arthritis correlate with Arthritis Impact Measurement Scales?. Clin. Exp. Rheumatol..

[6] Huang J., Fu X., Chen X., Li Z., Huang Y., Liang C. (2021). Promising Therapeutic Targets for Treatment of Rheumatoid Arthritis. Front. Immunol..

[7] Ono T., Hayashi M., Sasaki F., Nakashima T. (2020). RANKL biology: Bone metabolism, the immune system, and beyond. Inflamm. Regen..

[8] Okamoto K., Nakashima T., Shinohara M., Negishi-Koga T., Komatsu N., Terashima A., Sawa S., Nitta T., Takayanagi H. (2017). Osteoimmunology: The Conceptual Framework Unifying the Immune and Skeletal Systems. Physiol. Rev..

[9] Takegahara N., Kim H., Choi Y. (2022). RANKL biology. Bone.

[10] Yasuda H. (2021). Discovery of the RANKL/RANK/OPG system. J. Bone Miner. Metab..

[11] Udagawa N., Koide M., Nakamura M., Nakamichi Y., Yamashita T., Uehara S., Kobayashi Y., Furuya Y., Yasuda H., Fukuda C. (2020). Osteoclast differentiation by RANKL and OPG signaling pathways. J. Bone Miner. Metab..

[12] Wright H.L., McCarthy H.S., Middleton J., Marshall M.J. (2009). RANK, RANKL and osteoprotegerin in bone biology and disease. Curr. Rev. Musculoskelet. Med..

[13] Akiyama T. (2012). RANKL-RANK interaction in immune regulatory systems. World J. Orthop..

[14] Chino T., Draves K.E., Clark E.A. (2009). Regulation of dendritic cell survival and cytokine production by osteoprotegerin. J. Leukoc. Biol..

[15] Dostert C., Grusdat M., Letellier E., Brenner D. (2019). The TNF Family of Ligands and Receptors: Communication Modules in the Immune System and Beyond. Physiol. Rev..

[16] Sobacchi C., Menale C., Villa A. (2019). The RANKL-RANK Axis: A Bone to Thymus Round Trip. Front. Immunol..

[17] Irla M. (2021). RANK Signaling in the Differentiation and Regeneration of Thymic Epithelial Cells. Front. Immunol..

[18] Geusens P. (2012). The role of RANK ligand/osteoprotegerin in rheumatoid arthritis. Ther. Adv. Musculoskelet. Dis..

[19] Wu H., Khanna D., Park G., Gersuk V., Nepom G.T., Wong W.K., Paulus H.E., Tsao B.P. (2004). Interaction between *RANKL* and *HLA–DRB1* genotypes may contribute to younger age at onset of seropositive rheumatoid arthritis in an inception cohort. Arthritis Rheum..

[20] Assmann G., Koenig J., Pfreundschuh M., Epplen J.T., Kekow J., Roemer K., Wieczorek S. (2010). Genetic variations in genes encoding RANK, RANKL, and OPG in rheumatoid arthritis: A case-control study. J. Rheumatol..

[21] Tan W., Wu H., Zhao J., Derber L.A., Lee D.M., Shadick N.A., Conn D.L., Smith E.A., Gersuk V.H., Nepom G.T. (2010). A functional *RANKL* polymorphism associated with younger age at onset of rheumatoid arthritis. Arthritis Rheum..

[22] Zhang Y., Zhang H., Zhuang C., Liu R., Wei J. (2013). MSRA polymorphism is associated with the risk of rheumatoid arthritis in a Chinese population. Scand. J. Rheumatol..

[23] Ruyssen-Witrand A., Degboé Y., Cantagrel A., Nigon D., Lukas C., Scaramuzzino S., Allanore Y., Vittecoq O., Schaeverbeke T., Morel J. (2016). Association between *RANK*, *RANKL* and *OPG* polymorphisms with ACPA and erosions in rheumatoid arthritis: Results from a meta-analysis involving three French cohorts. RMD Open.

[24] Wielińska J., Kolossa K., Świerkot J., Dratwa M., Iwaszko M., Bugaj B., Wysoczańska B., Chaszczewska-Markowska M., Jeka S., Bogunia-Kubik K. (2020). Polymorphisms within the *RANK* and *RANKL* Encoding Genes in Patients with Rheumatoid Arthritis: Association with Disease Progression and Effectiveness of the Biological Treatment. Arch. Immunol. Ther. Exp..

[25] Yang H., Liu W., Zhou X., Rui H., Zhang H., Liu R. (2019). The association between RANK, RANKL and OPG gene polymorphisms and the risk of rheumatoid arthritis: A case-controlled study and meta-analysis. Biosci. Rep..

[26] Sánchez-Serrano C. (1996). Mestizaje e historia de la población en México (con un esbozo antropológico de los lacandones de Chiapas). Polimorfismo Génico (HLA) en Poblaciones Hispanoamericanas.

[27] Arnett F.C., Edworthy S.M., Bloch D.A., Mcshane D.J., Fries J.F., Cooper N.S., Healey L.A., Kaplan S.R., Liang M.H., Luthra H.S. (1988). The American Rheumatism Association 1987 revised criteria for the classification of rheumatoid arthritis. Arthritis Rheum..

[28] Prevoo M.L.L., van’t Hof M.A., Kuper H.H., Van Leeuwen M.A., Van De Putte L.B.A., Van Riel P.L.C.M. (1995). Modified disease activity scores that include twenty-eight-joint counts development and validation in a prospective longitudinal study of patients with rheumatoid arthritis. Arthritis Rheum..

[29] Fleischmann R.M., van der Heijde D., Gardiner P.V., Szumski A., Marshall L., Bananis E. (2017). DAS28-CRP and DAS28-ESR cut-offs for high disease activity in rheumatoid arthritis are not interchangeable. RMD Open.

[30] Cardiel M.H., Abello-Banfi M., Ruiz-Mercado R., Alarcon-Segovia D. (1993). How to measure health status in rheumatoid arthritis in non-English speaking patients: Validation of a Spanish version of the Health Assessment Questionnaire Disability Index (Spanish HAQ-DI). Clin. Exp. Rheumatol..

[31] Díaz-Toscano M.L., Olivas-Flores E.M., Zavaleta-Muñiz S.A., Gamez-Nava J.I., Cardona-Muñoz E.G., Ponce-Guarneros M., Castro-Contreras U., Nava A., Salazar-Paramo M., Celis A. (2014). Comparison of two assays to determine anti-citrullinated peptide antibodies in rheumatoid arthritis in relation to other chronic inflammatory rheumatic diseases: Assaying anti-modified citrullinated vimentin antibodies adds value to second-generation anti-citrullinated cyclic peptides testing. BioMed Res. Int..

[32] Miller S.A., Dykes D.D., Polesky H.F. (1988). A simple salting out procedure for extracting DNA from human nucleated cells. Nucleic Acids Res..

[33] Livak K.J. (1999). Allelic discrimination using fluorogenic probes and the 5′ nuclease assay. Genet. Anal. Biomol. Eng..

[34] Mencej S., Albagha O.M.E., Preželj J., Kocjan T., Marc J. (2008). Tumour necrosis factor superfamily member 11 gene promoter polymorphisms modulate promoter activity and influence bone mineral density in postmenopausal women with osteoporosis. J. Mol. Endocrinol..

[35] Leibbrandt A., Penninger J.M. (2008). RANK/RANKL: Regulators of immune responses and bone physiology. Ann. N. Y. Acad. Sci..

[36] Choi Y., Arron J.R., Townsend M.J. (2009). Promising bone-related therapeutic targets for rheumatoid arthritis. Nat. Rev. Rheumatol..

[37] Danks L., Komatsu N., Guerrini M.M., Sawa S., Armaka M., Kollias G., Nakashima T., Takayanagi H. (2016). RANKL expressed on synovial fibroblasts is primarily responsible for bone erosions during joint inflammation. Ann. Rheum. Dis..

[38] Wang C.-M., Tsai S.-C., Lin J.-C., Wu Y.-J.J., Wu J., Chen J.-Y. (2019). Association of Genetic Variants of *RANK*, *RANKL*, and *OPG* with Ankylosing Spondylitis Clinical Features in Taiwanese. Mediat. Inflamm..

[39] Shastry B.S. (2009). SNPs: Impact on gene function and phenotype. Methods Mol. Biol..

[40] Tanaka S., Tanaka Y. (2021). RANKL as a therapeutic target of rheumatoid arthritis. J. Bone Miner. Metab..

[41] Li J.-Y., Yu M., Tyagi A.M., Vaccaro C., Hsu E., Adams J., Bellido T., Weitzmann M.N., Pacifici R. (2018). IL-17 Receptor Signaling in Osteoblasts/Osteocytes Mediates PTH-Induced Bone Loss and Enhances Osteocytic RANKL Production. J. Bone Miner. Res..

[42] Luo G., Li F., Li X., Wang Z.-G., Zhang B. (2018). TNF-α and RANKL promote osteoclastogenesis by upregulating RANK via the NF-κB pathway. Mol. Med. Rep..

